# Transformational leadership competency: a cross-sectional study of medical university graduates in China

**DOI:** 10.1080/07853890.2023.2288307

**Published:** 2023-12-06

**Authors:** Yue Li, Sijie Li, Kun Yang, Yong Liu, Liying Chen, Jianjun Chen, Xiaochu Wen, Tingting Ji, Kefan Chen, Liyong Wu, Xunming Ji, Jie Lu

**Affiliations:** a Department of Education, Xuanwu Hospital, Capital Medical University, Beijing, China; b Beijing Key Laboratory of Hypoxic Conditioning Translational Medicine, Xuanwu Hospital, Center of Stroke, Beijing Institute of Brain Disorders, Capital Medical University, Beijing, China; c The National Center for Neurological Disorders, Xuanwu Hospital, Capital Medical University, Beijing, China; d Department of Education, Beijing Children’s Hospital, Capital Medical University, Beijing, China; e Department of Education, Beijing Anzhen Hospital, Capital Medical University, Beijing, China; f Department of Education, Beijing Friendship Hospital, Capital Medical University, Beijing, China; g Department of Education, Beijing Tongren Hospital, Capital Medical University, Beijing, China; h Department of Education, Beijing Chest Hospital, Capital Medical University, Beijing, China; i Department of Education, Beijing Chaoyang Hospital, Capital Medical University, Beijing, China; j Department of Neurology, Xuanwu Hospital, Capital Medical University, Beijing, China; k Beijing Institute of Brain Disorders, Capital Medical University, Beijing, China

**Keywords:** Transformational leadership competency, medical graduates, influencing variables

## Abstract

**Purpose:**

To explore the transformational leadership competency of graduates from one medical university in China and its influencing variables.

**Method:**

From 2020 to 2021, 851 medical graduates from seven hospitals affiliated with the Capital Medical University participated in this survey. The authors conducted a cross-sectional study to assess transformational leadership competency, particularly from three aspects, including values, Emotional Intelligence (EI) abilities, and behaviors using the socially responsible leadership scale (SRLS), emotionally intelligent leadership, and student leadership practices inventory (EILI and SLPI).

**Results:**

The SRLS scores were medium except for ‘controversy with civility’. The EILI scores were medium. The SLPI scores were high except for ‘enable others to act’ and ‘encourage the heart’. The influencing variables of SRLS, EILI, and SLPI were serving as student cadres, serving longer than two semesters (*p =* 0.01, 0.02 in EILI and SLPI), joining student organizations, participating in social practice, voluntary service (*p =* 0.001 in SLPI), in training classes for student cadres (*p =* 0.02, 0.01, 0.02 in SRLS, EILI, and SLPI), and attending lectures on leadership (except for indicated, *p* < 0.001). Regression analysis showed that attending lectures on leadership was associated with high SRLS, EILI, and SLPI scores (*p* = 0.04, SRLS; *p* < 0.001, others), and SRLS and EILI scores could affect SLPI score (*F* = 2674.44, *p* < 0.001, *R*^2^ = 0.86).

**Conclusions:**

Medical graduates’ transformational leadership competency at the Capital Medical University was medium measured from values, EI abilities, and behaviors. Group analysis indicated that knowledge learning, organizational involvement, and social/community involvement were associated with leadership capacity building, meanwhile, leaders’ values and EI abilities would affect their behaviors, suggesting medical graduates should undertake leadership training from both knowledge learning and practicing.

## 1. Introduction

With the rapid adoption of competency-based medical education [1], more attention should be paid to cultivating medical students’ competencies rather than specialized knowledge and skills. Leadership has been included as a sub-competency within two of their core competencies: professionalism/interpersonal and communication skills [2–4] by both the Accreditation Council for Graduate Medical Education (ACGME) and the China Elite Teaching Hospital Alliance for Resident Training. Clinicians need outstanding leadership to manage the entire diagnosis and treatment process, and researchers also need good leadership skills to organize efficient medical research activities [4]. The outline of the National Medium and Long-term Education Reform and Development Plan (2010–2020) clearly states that China should prioritize the cultivation of leadership skills among university students. It is not only an important component of quality education but also an urgent problem to be solved.

Leadership can be defined as the knowledge, values, abilities, and behaviors that help an individual to significantly contribute to or successfully engage in a role or task [5]. It also refers to a person’s ability to influence others positively [4]. The concept of ‘team leadership’-also known as shared leadership (emphasizing the importance of individual leadership competency improvement) is becoming more valued in healthcare education, the improvement of which will eventually contribute to a team’s success [6,7]. Valid leadership in medicine can improve team efficacy, patient outcome, and staff engagement and reduce physician burnout and medical errors [8]. The leadership competency of clinicians is becoming increasingly vital to meet patient-centered health care needs in the twenty-first century [9], especially in departments requiring more teamwork, like surgery, emergency medicine, and critical care.

There are many different leadership models, two broad types of them are ‘transactional’ and ‘transformational’. The latter is developed from the former and is based on inspiring individuals and forming teams to achieve goals with a clear vision and values [9]. Due to the continuous emergence of new technologies and evidence, the healthcare environment is particularly prone to change, and the necessity, inevitability, and complexity of change in healthcare made transformational leadership a key to medical leadership success [10]. The basis of it is the social change model, which was developed by the University of North Carolina Higher Education Research Institute in 1993, and the key assumption in the model is that effective leadership is about positive social change on behalf of others and society [11].

In recent years, an increasing number of studies have measured the transformational leadership competency of medical undergraduates. Most of them adapted one instrument, and some compared students’ leadership competency before and after training, while others focused on students of a sub-major, such as pharmacy or physical therapy [12–14]. We assessed the medical graduates’ leadership competency from values, EI abilities, and behaviors based on leadership definition, and group analysis showed knowledge learning, organizational involvement, and social/community involvement were associated with leadership competency improving. This fills a gap in leadership research of medical graduates in China.

## 2. Methods

This study adopted a scale survey method that conforms to the ethical principles of medical research in the Declaration of Helsinki. We used the translated version [15] of the SRLS Revised Version 2 [12,14,16] for values assessment, EILI [13,17] for EI abilities assessment, and SLPI [12,18] for behaviors assessment. A 5-point scale Likert was used to classify the options: for SRLS, 1 = strongly disagree, 5 = in full agreement; and for EILI and SLPI, 1 = never, 5 = always. The Kaiser–Meyer–Olkin (KMO) measure and the Bartlett’s test for sphericity were used to demonstrate the appropriateness of the utilization of factor analysis and Cronbach’s *α* was used to test the reliability of the question.

### 2.1. Setting and sample

From 2020 to 2021, a completely randomized digital table sampling method was conducted on 2431 graduate students of 7 from 23 (30.43%) affiliated hospitals of CMU using student numbers, and a total of 851 (35.01% of the chosen hospitals and 17.70% of the whole students) students participated in this survey using Questionnaire Network software (Zhongyan Technology, China, Shanghai, and Suzhou). Completely randomized digital tables were also conducted in choosing the hospitals, finally, 3 of 15 (40.74% of the whole students) specialized hospitals and 4 of 8 (72.81% of the whole students) comprehensive hospitals were chosen. Among these, 268 were from Xuanwu Hospital, 243 from Beijing Children’s Hospital, 103 from Beijing Anzhen Hospital, 90 from Beijing Friendship Hospital, 61 from Beijing Tongren Hospital, 45 from Beijing Chest Hospital, and 41 from Beijing Chaoyang Hospital. All participants completed the survey and provided written informed consent. The inclusion criteria were graduates willing to participate in the study and answer the question honestly, and those who did not obey this rule were excluded. This study was approved by the Xuanwu Hospital Review Board. The 268 graduates from Xuanwu Hospital completed the survey from 2020 to 2021 for the preliminary experiment, and for others, they completed the survey in 2021, all of which were performed from May to September.

### 2.2. Survey questionnaire of some sociodemographic characteristics

This study included demographic information of the graduates, such as gender, household registration district, grade, whether the only child of a family, degree level/category, and political status. The social activities of the participants were divided into organizational involvement like whether they served as student cadres, student cadre position, and duration, whether they joined student organizations, and social/community involvement like whether they participated in social practice, voluntary service, and whether they held a part-time job. The leadership learning status of the participants was divided into leadership curricula learning (systemic learning-university based), student cadres training (strengthened learning-student affairs department based), and taking lectures on leadership (occasionally learning-interest based).

### 2.3. Assessment of transformational leadership

#### 2.3.1. Values assessment

The SRLS is based on the social change model of leadership development, it assesses values of leadership skills, known as the ‘7 C’s of leadership development for social change [19,20], comprising 68 questions evaluating individual values-including ‘consciousness of self’, ‘congruence’, ‘commitment’, group values-including ‘collaboration’, ‘common purpose’, ‘controversy with civility’ and community values-including ‘citizenship’. ‘Change’ was added as the eighth value to the model [12,21]. The total score was 340 points, and Cronbach’s alpha coefficient was 0.966, Kaiser–Meyer–Olkin (KMO) measure coefficient was 0.983 (*x*^2^ = 49489.556, *p* < 0.001). The original scale item was disordered to overcome the common method bias caused by the social approval effect, and a reverse question was introduced [22]. Each dimension can be categorized as high, medium, or low (Supplemental Table 1) [12,16].

**Table 1.** General characteristics and statistics among groups of 851 medical graduates in Capital Medical University studied in 2020–2021.

Category/variable	*N* (percentage /%)	SRLS		EILI		SLPI	
		Mean (*SD*)	*t* or *F/P*	Mean (*SD*)	*t* or *F/P*	Mean (*SD*)	*t* or *F/P*
Demographic characteristics							
Gender (F vs. M)			0.26/0.80		0.79/0.43		0.11/0.92
Male	287 (33.73)	278.70 (36.49)		100.0 (15.87)		126.06 (20.24)	
Female	564 (66.27)	278.06 (33.85)		99.13 (14.99)		125.91 (18.98)	
Household registration district (R vs. U)			1.62/0.11		1.74/0.08		1.46/0.15
Urban area	575 (67.57)	279.61 (34.35)		100.04 (15.51)		126.63 (19.51)	
Rural area	276 (32.43)	275.49 (35.45)		98.13 (14.76)		124.57 (19.13)	
Grade (F vs. S)			−1.38/0.17		−0.26/0.79		0.32/0.75
Senior	277 (32.55)	275.90 (36.51)		99.22 (15.82)		126.27 (20.20)	
Freshmen	574 (67.45)	279.42 (33.83)		99.51 (15.74)		125.82 (19.02)	
The only child (Y vs. N)			1.88/0.06		1.56/0.12		1.20/0.23
Yes	461 (54.17)	280.32 (36.23)		100.17 (16.09)		126.69 (20.38)	
No	390 (45.83)	275.85 (32.77)		98.54 (14.25)		125.11 (18.17)	
Degree level (D vs. M)			−1.02/0.31		−1.26/0.21		−1.63/0.10
Master	599 (70.39)	277.49 (34.46)		99.00 (15.42)		125.26 (19.30)	
Doctor	252 (29.61)	280.14 (35.39)		100.44 (14.94)		127.63 (19.57)	
Degree category (P vs. S)			1.40/0.16		0.67/0.51		1.04/0.30
Scientific degree	247 (29.02)	280.87 (35.98)		99.97 (15.98)		127.04 (20.14)	
Professional degree	604 (70.98)	277.21 (34.19)		99.19 (15.91)		125.52 (19.09)	
Political party members (Y vs. N)			2.26/0.02*		0.73/0.47		0.43/0.67
Yes	245 (28.79)	282.49 (31.86)		100.02 (15.23)		126.42 (18.94)	
No	606 (71.21)	276.57 (35.73)		99.18 (15.32)		125.78 (19.60)	
Social activities							
Organizational involvement							
Student cadre (Y vs. N)			6.61/<0.001*		6.34/<0.001*		5.30/<0.001*
Yes	488 (57.34)	285.09 (34.63)		102.32 (13.25)		129.06 (16.84)	
No	363 (42.66)	269.10 (37.78)		95.52 (16.92)		121.80 (21.73)	
Position (secretary as baseline)			3.13/0.045*		3.45/0.03*		0.89/0.41
Secretary	359 (73.56)	283.03 (36.76)		101.44 (13.97)		128.48 (16.72)	
Chairman	77 (15.78)	291.35 (31.77)		105.67 (14.53)		131.19 (18.10)	
Clerkship	52 (10.66)	290.03 (27.89)	0.03^#^	103.38 (12.67)	0.01^#^	129.90 (15.78)	
Duration of act as student cadre (<1 semester as baseline)			9.02/<0.001*		5.24/0.01*		4.10/0.02*
<1 semester	57 (11.68)	280.53 (32.42)		101.91 (13.36)		128.44 (17.15)	
1–2 semesters	203 (41.6)	279.44 (32.99)		100.21 (13.65)		126.71 (16.55)	
Longer than 2 semesters	228 (46.72)	291.27 (26.62)	0.02*/<0.001^#^	104.30 (12.59)	0.001^#^	131.31 (16.80)	0.01^#^
Join student organizations (Y vs. N)			7.72/<0.001*		6.42/<0.001*		5.62/<0.001*
Yes	337 (39.60)	288.81 (29.35)		103.32 (13.14)		130.31 (16.66)	
No	514 (60.40)	271.36 (36.26)		96.86 (16.45)		123.11 (20.53)	
Social/community involvement							
Social practice (Y vs. N)			4.14/<0.001*		3.99/<0.001*		3.64/<0.001*
Yes	750 (88.13)	280.47 (32.84)		100.35 (14.48)		127.03 (18.46)	
No	101 (11.87)	261.94 (43.31)		92.50 (19.52)		118.02 (23.97)	
Voluntary service (Y vs. N)			3.89/<0.001*		3.99/<0.001*		3.42/0.001*
Yes	748 (87.90)	280.31 (33.11)		100.37 (14.45)		126.98 (18.50)	
No	103 (12.10)	263.49 (42.15)		92.56 (19.11)		118.61 (23.84)	
Part-time job (Y vs. N)			2.17/0.03*		1.89/0.06		2.69/0.01*
Yes	481 (56.52)	280.54 (32.64)		100.30 (14.36)		100.30 (14.36)	
No	370 (43.48)	275.33 (37.14)		98.27 (16.43)		98.27 (16.43)	
Leadership knowledge learning							
Leadership curricula (Y vs. N)			1.71/0.09		1.15/0.25		1.14/0.26
Yes	59 (6.93)	285.71 (33.21)		101.63 (14.93)		128.73 (18.49)	
No	792 (93.07)	277.72 (34.81)		99.26 (15.31)		125.76 (19.46)	
Training for student cadres (Y vs. N)			2.28/0.02*		2.77/0.01*		2.27/0.02*
Yes	130 (26.64)	290.30 (28.66)		105.06 (12.42)		131.92 (16.49)	
No	358 (73.36)	283.20 (31.14)		101.33 (13.41)		128.03 (16.87)	
Lectures on leadership (Y vs. N)			5.66/<0.001*		5.30/<0.001*		4.69/<0.001*
Yes	181 (21.27)	291.01 (37.81)		104.68 (13.65)		131.90 (16.96)	
No	670 (78.73)	274.83 (34.96)		98.00 (15.42)		124.36 (19.72)	

^*^ Indicates comparison to baseline, *p* < 0.05; ^#^Indicates from up to down in the same group, last *vs.* middle, *p* < 0.05. Statistically significant differences in the scores of the three scales between different groups are presented in Supplemental Table 1.

#### 2.3.2. EI abilities assessment

The EILI is based on the emotionally intelligent leadership model, it assesses emotional intelligence (one of the core abilities of transformational leadership [23]) of leadership skills, comprising 24 questions assessed from the three dimensions of consciousness-’context’, ‘self’, and ‘others’-with a total of 120 points, a Cronbach’s alpha coefficient of 0.961 and a Kaiser–Meyer–Olkin (KMO) measure coefficient of 0.976 (*x*^2^ = 22380.668, *p* < 0.001). Each dimension can be categorized as high, medium, or low (Supplemental Table 1) [17].

#### 2.3.3. Behaviors assessment

The SLPI is based on a leadership challenge program developed by the social change model and assesses five key practices of 30 behaviors of leadership skills [14]-’model the way’, ‘inspire a shared vision’, ‘challenge the process’, ‘enable others to act’, and ‘encourage the heart’-with a total of 150 points. Cronbach’s alpha coefficient was 0.985 and Kaiser-Meyer-Olkin (KMO) measure coefficient was 0.985 (*x*^2^ = 31342.083, *p* < 0.001). Each dimension can be categorized as high, medium, or low (Supplemental Table 1) [18,24].

### 2.4. Data analysis

The Kolmogorov–Smirnov test, Q–Q, and P–P plots were used to confirm normality for continuous variables. All normally distributed continuous variables are reported as mean(standard deviation). When the data were normally distributed and the variances were homogeneous, *t*-test or analysis of variance was used for univariate analysis. Categorical variables are represented by frequency and percentage. Binary logistic regression was performed for univariate or multivariate analysis. The cut-off value of binary logistic regression was 319 for SRLS, which was revised by the authors due to different versions (Version 2 *vs.* 3) [12,16], 105 for EILI [17], and 125 for SLPI [18,24]. We hypothesized that SRLS and EILI scores can affect SLPI scores and then tested this hypothesis by multivariate linear regression with SLPI scores as the dependent variables and SRLS and EILI scores as independent variables. Statistical significance was set at 0.05 (2-sided). The data were analyzed using SPSS 25.0 for Windows (IBM Corp, Armonk, NY, USA).

## 3. Results

### 3.1. Sociodemographic characteristics of the participants

Among the 851 medical graduates, 66.27% were female, 67.57% came from urban areas, 67.45% were freshmen, and 54.17% were only child of a family. 70.39% were masters and 70.98% were professional degree students, which meant they were trained to be doctors. On the other hand, the scientific degree students meant that they were trained to be scientists. Of these, 28.79% were political party members. A total of 57.34% served as student cadres (which meant they were appointed leaders by the student affairs department). Of the student cadres, 73.56% served as secretaries, 46.72% served longer than two semesters, and 26.64% took training classes (15.28% of all participants). In total, 39.6% joined student organizations, 88.13% participated in social practices, 87.9% participated in voluntary services, and 56.52% held part-time jobs. A total of 6.93% took leadership curricula and 21.27% attended lectures on leadership (Table 1).

### 3.2. SRLS EILI and SLPI mean scores and their levels of medical graduates

The scores of SRLS were 278.27 (34.74), and of the dimensions of ‘consciousness of self’, ‘congruence’, ‘commitment’, ‘collaboration’, ‘common purpose’, ‘citizenship’, and ‘change’ were 35.19 (4.89), 28.84 (4.07), 25.27 (3.56), 33.46 (4.79), 38.39 (5.31), 33.76 (5.01), 39.75 (5.58), respectively, which were medium. The scores of ‘controversy with the civility’ in SRLS were 43.6 (5.16), which were low. The scores of EILI were 99.42 (15.29), and of the dimensions of ‘consciousness of context, self, and others’ were 32.12 (5.53), 33.82 (4.95), 33.48 (5.38), respectively, which were medium. The scores of SLPI were 125.96 (19.4), and of dimensions of ‘model the way’, ‘inspire a shared vision’, ‘challenge the process’ were 25.26 (3.99), 25.2 (4.09), 24.85 (4.15), which were high. The scores of ‘enable others to act ‘and ‘encourage the heart’ in SLPI were 25.31 (3.74) and 25.36 (3.99), respectively, at a medium level (Supplemental Table 1).

### 3.3. Univariate analysis of the influencing variables of SRLS, EILI, and SLPI mean scores

Students who served as student cadres (*n* = 488, 57.34%), and served longer than two semesters (*n* = 228, 46.72%, *p =* 0.01 and 0.02 in EILI and SLPI), joined student organizations (*n* = 337, 39.6%), participated in social practice (*n* = 750, 88.13%), in voluntary service (*n* = 748, 87.9%, *p =* 0.001 in SLPI), and in the training classes for student cadres (*n* = 130, 26.64%, *p =* 0.02, 0.01, 0.02 in SRLS, EILI and SLPI), and attended lectures on leadership (*n* = 181, 21.27%) scored higher than their counterparts in SRLS, EILI and SLPI (except for indicated, *p* < 0.001). Political party members (*n* = 245, 28.79%, *p* = 0.02) scored higher than others in SRLS. The chairman (*n* = 77, 15.78%) scored higher than others in SRLS (*p* = 0.045) and EILI (*p* = 0.03). Students who held part-time jobs (*n* = 481, 56.52%) scored higher than their counterparts in SRLS (*p* = 0.03) or SLPI (*p* = 0.01).

### 3.4. Independent influencing variables of SRLS, EILI, and SLPI mean scores

The single factor logistic regression analysis picked up factors for multiple factor logistic regression, for the ‘duration of act as student cadre’ variable, after comparison, we concluded the model was better if we ruled it out (Table 2). The multiple factor logistic model was statistically significant (for each model, *p* < 0.001), *χ*^2^ = 34.56, 50.38, 45.38 for SRLS, EILI, and SLPI, respectively. For the SRLS scale, only child were 1.77 times more likely to score higher than their counterparts, participants who joined student organizations were 1.74 times more likely to score higher, and participants who attended lectures on leadership were 1.65 times more likely to score higher. For the EILI scale, doctors were 1.43 times more likely to score higher than masters, participants who joined student organizations were 1.49 times more likely to score higher, and participants who took lectures on leadership were 1.82 times more likely to score higher. For the SLPI scale, doctors were 1.45 times more likely to score higher than masters, participants who joined student organizations were 1.33 times more likely to score higher, and participants who attended lectures on leadership were 1.78 times more likely to score higher (Table 3).

**Table 2.** Univariate logistic regression results of 851 medical graduates in Capital Medical University studied in 2020–2021.

Variable	SRLS		EILI		SLPI	
	OR (95% CI)	*p*	OR (95% CI)	*p*	OR (95% CI)	*p*
Demographic characteristics						
Gender (F vs. M)	0.78 (0.51–1.19)	0.24	0.78 (0.59–1.05)	0.10	0.83 (0.62–1.10)	0.19
Household registration district (R vs. U)	1.41 (0.88–2.24)	0.15	1.41 (1.04–1.90)	0.03*	1.35 (1.01–1.80)	0.04*
Grade (F vs. S)	1.14 (0.73–1.78)	0.57	0.86 (0.64–1.15)	0.30	1.03 (0.77–1.37)	0.86
The only child (Y vs. N)	0.54 (0.35–0.84)	0.01*	0.69 (0.53–0.92)	0.01*	0.81 (0.62–1.06)	0.13
Degree level (D vs. M)	1.03 (0.65–1.62)	0.91	0.75 (0.55–1.01)	0.06	0.72 (0.54–0.97)	0.03*
Degree category (P vs. S)	1.11 (0.70–1.76)	0.66	0.82 (0.61–1.11)	0.20	0.78 (0.58–1.05)	0.10
Political party members (Y vs. N)	1.32 (0.85–2.04)	0.22	1.08 (0.79–1.46)	0.64	0.97 (0.72–1.31)	0.84
Social activities						
Organizational involvement						
Student cadre	1.95 (1.25–3.06)	0.01*	1.71 (1.28–2.27)	<0.001*	1.74 (1.32–2.29)	<0.001*
Position (chairman as baseline)	0.98 (0.40–2.40)	0.97	0.62 (0.31–1.27)	0.19	0.58 (0.29–1.20)	0.14
Duration of act as student cadre (1–2 semesters as baseline)	1.21 (0.71–2.06)	0.48	1.46 (1.00–2.14)	0.05	1.75 (1.19–2.56)	0.01*
Join student organizations (Y vs. N)	2.27 (1.49–3.44)	<0.001*	1.87 (1.41–2.48)	<0.001*	1.73 (1.31–2.29)	<0.001*
Social/community involvement						
Social practice (Y vs. N)	3.69 (1.33–10.25)	0.01*	1.79 (1.12–2.84)	0.01*	1.76 (1.14–2.72)	0.01*
Voluntary service (Y vs. N)	2.96 (1.17–7.44)	0.02*	1.84 (1.16–2.93)	0.01*	1.74 (1.13–2.67)	0.01*
Part-time job (Y vs. N)	1.30 (0.85–1.99)	0.22	1.19 (0.90–1.57)	0.23	1.18 (0.90–1.55)	0.23
Leadership knowledge learning						
Leadership curriculum (Y vs. N)	1.34 (0.64–2.81)	0.44	1.52 (0.90–2.59)	0.12	1.49 (0.88–2.54)	0.14
Training for student cadres (Y vs. N)	1.54 (0.90–2.62)	0.11	1.45 (0.97–2.17)	0.07	1.45 (0.97–2.18)	0.07
Lectures on leadership (Y vs. N)	2.12 (1.36–3.32)	0.001*	2.20 (1.58–3.08)	<0.001*	2.13 (1.52–2.98)	<0.001*

The univariate logistic regression analysis picked up factors for multiple factor logistic regression, cut off value equals to *p* < 0.05, indicated as ‘*’ in Table 2. For the duration of act as student cadre variable, after comparison, we concluded the model was better if we rule it out.

**Table 3.** Multivariate logistic regression results of 851 medical graduates in Capital Medical University studied in 2020–2021.

Variable	SRLS		EILI		SLPI	
	OR (95% CI)	*B/p*	OR (95% CI)	*B/p*	OR (95% CI)	*B/p*
Household registration district (R *vs.* U)	0.97 (0.57–1.68)	−0.03/0.92	0.81 (0.57–1.17)	−0.21/0.26	0.77 (0.55–1.10)	−0.26/0.15
The only child (Y *vs.* N)	1.77 (1.07–2.91)	0.57/0.03*	1.30 (0.93–1.81)	0.26/0.13	1.07 (0.77–1.48)	0.07/0.68
Degree level (D *vs.* M)	1.02 (0.64–1.62)	0.02/0.94	1.43 (1.05–1.96)	0.36/0.02*	1.45 (1.07–1.97)	0.37/0.02*
Student cadre (Y *vs.* N)	1.19 (0.71–2.02)	0.18/0.51	1.20 (0.86–1.68)	0.18/0.29	1.30 (0.94–1.80)	0.27/0.11
Join student organizations (Y *vs.* N)	1.74 (1.07–2.83)	0.55/0.03*	1.49 (1.07–2.07)	0.40/0.02*	1.33 (0.97–1.85)	0.29/0.08*
Social practice (Y *vs.* N)	2.29 (0.75–6.97)	0.83/0.15	1.29 (0.74–2.23)	0.25/0.37	1.33 (0.79–2.22)	0.28/0.29
Voluntary service (Y *vs.* N)	1.50 (0.55–4.14)	0.41/0.43	1.28 (0.74–2.21)	0.25/0.38	1.21 (0.73–2.02)	0.19/0.46
Lectures on leadership (Y *vs.* N)	1.65 (1.03–2.65)	0.50/0.04*	1.82 (1.28–2.58)	0.60/<0.001*	1.78 (1.26–2.53)	0.58/<0.001*

The multivariate logistic model is statistically significant (for each model, *p* < 0.001), *x*^2^ = 34.56, 50.38, 45.38 for SRLS, EILI, and SLPI, respectively. The only child were 1.77 times more likely to score high on the SRLS scale than non-only child. Participants who joined student organizations were 1.74 times more likely to score high on the SRLS scale than those who did not and so on.

### 3.5. Predictive ability of SRLS and EILI scores for SLPI score

The scores of the SLPI survey were taken as dependent variables, and those of the SRLS and EILI as independent variables. SLPI scores were positively affected by SRLS and EILI scores. As shown in Table 4, the regression equation was SLPI = −1.86 + 0.13*SRLS + 0.93*EILI (*F* = 2674.44, *p* < 0.001, *R*^2^ = 0.86). *B* is the constant term in the regression equation. The regression coefficient of SRLS was 0.13, whereas that of EILI was 0.93. Both were statistically significant (*p* < 0.001). The variance inflation factor (VIF) was 3.13, indicating that collinearity was within the acceptable range (VIF < 5).

**Table 4.** The multivariate linear regression results of 851 medical graduates in Capital Medical University studied in 2020–2021.

Independent variable	Partial regression coefficient		Standard regression coefficient beta	*t*	*p*	Collinearity statistics	
	*B*	Standard error				Allowance	VIF
Constant term	−1.86	1.99		−0.94	0.35		
SRLS	0.13	0.01	0.23	10.19	<0.001*	0.32	3.13
EILI	0.93	0.03	0.73	32.51		0.32	3.13

The regression equation could be established as SLPI = −1.86 + 0.13*SRLS + 0.93*EILI (*F* = 2674.44, *p* < 0.001, *R*^2^ = 0.86).

## 4. Discussion

We conducted a large-scale, cross-sectional, multi-institutional transformational leadership competency survey of medical graduates in China from values, EI abilities, and behaviors. It was found that medical graduates’ leadership competency at our university was medium, and the influencing variables of them were knowledge learning (taking training classes for student cadres or lectures on leadership), organizational involvement (serving as student cadres, serving longer than two semesters, joining student organizations) and social/community involvement (participating in social practice, voluntary service). Regression analysis showed that knowledge learning (attending lectures on leadership) was associated with high SRL, EIL, and SLP levels, meanwhile, SRL and EIL levels could affect SLP levels.

In other studies mentioned in the introduction, the SRLS scores were medium [12] to high [25] and EILI mean scores were medium [13], the mean scores of SLPI from a national sample in the data of 1.1 million were medium [26]. One study showed that the SLPI mean scores were low before and medium after taking leadership curricula [14]. In contrast, the leadership competency of medical graduates at CMU was medium, relatively weak in ‘controversy with civility’, ‘enable others to act’, and ‘encourage the heart’. This implied that they might not be good at influencing other people through respectful conflict [11]. And more targeted strategies might be developed to fill this gap. Hamann [27] determined that the sociocultural interactions of students with their peers and community service, as well as their grade averages, gender, and leadership training before university, were important determinants of the SRL capacities of college students. This was consistent with ours, except for the grade and gender. Participants’ self-assessment of 15 leadership competency skills (including self-management, authenticity/sincerity, conflict management, group/team guidance, dealing with people-skills of EILI) significantly improved 6-month later following the leadership knowledge training [28]. Posner [29] reported that formal education and exposure to leadership opportunities resulted in more frequent use of leadership behaviors. In our study, we only selected a few variables of sociodemographic characteristics influencing leadership competency, almost every variable of social activities would influence an individual’s leadership competency, and the situation was the same when it came to leadership learning status.

According to the research results of Dugan and Komives, college students who participated in student organizations had higher average scores on eight dimensions of SRL than those who did not [30]. The EIL level of college students who participated in student organizations was higher than that of college students who did not [31]. So act as a student cadre [30,31]. Our group comparison and regression analysis consistently suggested that taking social activity was associated with SRL, EIL, and SLP leadership competency improvement. The more activities they joined, the more they communicated, and the more they would learn to mobilize, organize, and create. Being chairman influenced SRL and EIL levels, the chairman could have more chances to act as a leader, and this would contribute to value shaping and EI improvement. In the regression analysis of Betül Sönmez, the participation of students in student clubs was effective in their high SRLS scores (2.3 times) [12], ours was 1.74 times. This highlighted the importance of experiential learning. Experiential learning means to construct knowledge and meaning from real-life experience, whose foundation is social interactions and whose core condition is active participation [32]. Among them, the service-based learning fits perfectly with the culture and demands of medical education and is a good way to develop leadership skills [33,34]. Residency is the ideal stage to expose young physicians to leadership capacity building [35]. Considering that 70.98% of our participants were future clinical practitioners (professional degree doctors and masters), the method of leadership implementation during resident training is worth investigating. Since the mid-1990s, the American resident training model has shown a trend of emphasizing primary health care, the contents and methods of it are more often trained in community health institutions (One good example of service-based learning) [36].

The student leadership challenge model regards education: learning in formal training as one of the three important ways of leadership learning [37]. Our group and regression analyses also suggested knowledge learning could play a role in leadership capacity building, which emphasized the importance of consciousness of leadership and leadership identity development [38,39]. Leadership knowledge topics could include managerial skills, risk management, team collaboration, conflict resolution, communication and interpersonal skills, feedback, and professionalism [40,41]. For us, only 6.93% of participants took leadership curricula (too few positive samples made the comparison not statistically significant), 26.24% took training classes (15.28% of all participants), and 21.27% attended lectures on leadership, which is lower than the national survey proportion (28.5%) [42]. Actions should be taken to improve this situation.

SRLS focuses on change and is influenced by individual, group, and social values [11]. The performance evaluation rubric of it focuses on professionalism, communication, critical thinking, and problem-solving. EI is an important underlying factor affecting leadership behavior [43,44]. The Hay Group claimed that EI accounts for more than 85% of the exceptional performance of top leaders [45]. Emotions affect an individual’s leadership behaviors by influencing their thoughts, decisions, and behaviors [13]. A balance of all leadership behaviors in SLPI may lead to a person act as a transformational leader most effectively [14]. Stogdill and Bentz revealed that leadership competency is positively related to surgency, emotional stability, sense of responsibility, and easygoing [46,47]. Inspired by this, we hypothesized that leader’s values and EI abilities could affect their behaviors, which was confirmed by our regression analysis. Even though causation cannot be inferred, a predictive model had been built by us, 86% of the variations could be explained by the model, suggesting a strong relationship.

A limitation of this study was that the scales used were self-reporting. This can introduce bias, as participants might overestimate or underestimate their competencies or be influenced by social desirability. It was a non-intervention cross-sectional study, this design does not allow for assessment of changes in leadership competencies over time or causal inferences. Furthermore, other variables like family background, prior experiences or cultural factors might be influencing leadership competencies, which were not assessed by us. There might be other aspects of measurements of transformational leadership competencies that were not covered by us. The scales were not designed for medical students and the leadership competencies we measured in an academic context, which might not translate directly to leadership competencies required in medical practice or other real-world scenarios. If we did this survey in a competitive field, there might be a higher likelihood of them responding in a manner they perceive as favorable. Last but not least, we did this survey of a specific group of samples in specific regions, whether the theoretical and managerial implications could be applied to other groups and other regions remains to be explored.

## 5. Conclusions

The transformational leadership competency of medical graduates at CMU was medium measured from values, EI abilities, and behaviors. Group analysis indicated that knowledge learning, organizational involvement, and social/community involvement were associated with leadership capacity building. Leaders’ values and EI abilities could affect their behaviors. Quality leadership education might be essential to improve healthcare practices and research outcomes.

## Supplementary Material

Supplemental Material

## Data Availability

Additional data can be accessed *via* request at [URL] with doi:10.5281/zenodo.7847746.
