# Supplementary material for: Transformational leadership competency: a cross-sectional study of medical university graduates in China
Source: Ann Med. 2023 Dec 6;55(2):2288307. doi: 10.1080/07853890.2023.2288307 (PMC10836284; doi:10.1080/07853890.2023.2288307)
Supplement: Supplemental Material [file IANN_A_2288307_SM1269.docx]

**Supplemental Table 1. Classification of Socially Responsible, Emotionally Intelligent and Leadership Practices Scale**

| Scale name |  | High | Medium | Low | Mean(SD) |
| --- | --- | --- | --- | --- | --- |
| SRLS | Consciousness of self | >37 | 32-37 | <32 | 35.19(4.89) |
|  | Congruence | >37 | 32-37 | <28 | 28.84(4.07) |
|  | Commitment | >29 | 24-29 | <24 | 25.27(3.56) |
|  | Collaboration | >36 | 30-36 | <30 | 33.46(4.79) |
|  | Common Purpose | >40 | 34-40 | <34 | 38.39(5.31) |
|  | Controversy with Civility | >64 | 53-64 | <53 | 43.60(5.16) |
|  | Citizenship | >35 | 29-35 | <29 | 33.76(5.01) |
|  | Change | >41 | 35-41 | <35 | 39.75(5.58) |
|  | total | >319 | 319-265 | <265 | 278.27(34.74) |
| EILI | Consciousness of context/self/others | 35-40 | 26-34 | 8-25 | 32.12(5.53)/33.82(4.95)/33.48(5.38) |
|  | total | 105-120 | 78-102 | 24-75 | 99.42(15.29) |
| SLPI | Model the way | 25-30 | 21-24 | 17-20 | 25.26(3.99) |
|  | Inspire a shared vision | 24-30 | 19-23 | 14-18 | 25.20(4.09) |
|  | Challenge the process | 24-30 | 19-23 | 14-18 | 24.85(4.15) |
|  | Enable others to act | 26-30 | 23-25 | 18-22 | 25.31(3.74) |
|  | Encourage the heart | 26-30 | 22-25 | 17-21 | 25.36(3.99) |
|  | total | 125-150 | 104-120 | 80-99 | 125.96(19.40) |

Supplemental Table 1 shows the scores of SRLS, EILI, and SLPI and their different dimensions and the classification details.
